# Examining the Influence of Shift Length on Nurse Fatigue, Patient Care, Quality of Life, and Work-Life Dynamics in a Tertiary Hospital in Oman: Comparative Study

**DOI:** 10.1155/nrp/7946997

**Published:** 2025-07-07

**Authors:** Asma Al Yahyaei, Eman Al Rabaani, Rahaf Alkasbi, Yumna Alhashmi, Ibrahim Al Hatmi

**Affiliations:** College of Nursing, Sultan Qaboos University, Seeb, Oman

**Keywords:** 8-h shifts, 12-h shifts, nurse fatigue, patient safety, quality of care, shift length

## Abstract

**Background:** The adoption of 12-h shifts in response to workforce shortages has raised questions about their impact on nurses' well-being and patient care quality. While international studies offer mixed findings, little is known about these effects in the Omani context.

**Aim:** To compare the effects of 8-h and 12-h shifts on nurse fatigue, quality of life, satisfaction, turnover intention, and perceived patient care in a tertiary hospital in Oman.

**Methods:** A comparative cross-sectional study was conducted with 100 nurses from Sultan Qaboos University Hospital using self-administered surveys. Descriptive statistics, *t*-tests, and chi-square tests were performed to compare outcomes across shift types. A multiple regression analysis was also conducted to assess predictors of quality of life, controlling for shift type, age, gender, marital status, satisfaction, fatigue, and unit assignment.

**Results:** Nurses working 12-h shifts reported significantly higher levels of fatigue (*p* < 0.001; Cohen's *d* = 0.82) but also reported higher quality of life (*p* < 0.001; *d* = 0.91) and greater satisfaction with their shift length (*p*=0.001; *d* = 0.72). No significant differences were found in turnover intention. Patient falls were more frequently reported among 12-h shift nurses, while other safety incidents showed no significant variation. A multiple regression model predicting QOL was significant (*F*(16, 83) = 9.64, *p* < 0.001, adjusted *R*^2^ = 0.583). Satisfaction with shift was the strongest positive predictor (*β* = 0.432, *p* < 0.001, 95% CI [0.117, 0.287]), while working a 12-h shift had a significant negative association with QOL (*β* = −0.232, *p*=0.004, 95% CI [−0.603, −0.117]). Marital status showed a marginally significant positive effect (*β* = 0.180, *p*=0.051, 95% CI [−0.001, 0.555]).

**Conclusion:** Despite increased fatigue, nurses working 12-h shifts reported higher quality of life, possibly due to more days off and enhanced work-life balance. However, elevated fatigue and fall rates warrant caution. The findings underscore the importance of implementing flexible and individualized scheduling policies rather than universally adopting 12-h shifts. Further longitudinal studies are needed to explore long-term effects of shift length on nurse and patient outcomes.

**Summary**

• What is already known about this topic?
◦ Nurse fatigue is a critical concern in healthcare settings due to its potential impact on patient safety and quality of care.
◦ Previous studies have provided mixed findings regarding the association between shift length (8 h vs. 12 h shifts) and nurse fatigue, with limited research conducted in Oman.
◦ The effects of 12-h shifts on nurse outcomes and quality of care during the COVID-19 pandemic remain largely unexplored.
• What this paper adds:
◦ This study comprehensively evaluates the impact of 12-h shifts on nurse fatigue, quality of care, turnover intention, and satisfaction compared to 8-h shifts at Sultan Qaboos University Hospital (SQUH) in Oman.
◦ Findings reveal no significant differences in nurse fatigue, quality of care, turnover intention, or satisfaction between nurses working 8- and 12-h shifts, providing valuable insights specific to the Omani context.
◦ The research fills a critical gap in understanding the consequences of implementing 12-h shifts during the COVID-19 pandemic, shedding light on the unique challenges and benefits associated with this shift length in Oman.
• The implications of this paper:
◦ Policymakers and healthcare managers can utilize the findings to inform decisions about nursing shift lengths, ensuring considerations for patient safety, care quality, and nurse retention.
◦ The study highlights the feasibility of implementing 12-h shifts as an alternative to address nursing shortages during emergencies without compromising nurse outcomes or patient care quality.
◦ Future research can build upon these findings to further investigate the long-term effects of shift length on nurses' well-being, patient outcomes, and healthcare system efficiency in Oman and other similar contexts.

## 1. Introduction

Globally, healthcare systems rely on shift work to provide continuous patient care, with 8- and 12-h shifts being most common. The COVID-19 pandemic accelerated the adoption of 12-h shifts in many healthcare settings, including SQUH in Oman. This shift aimed to address workforce shortages due to staff quarantine, illness, and the increased acuity of patients. However, the evidence surrounding the effects of shift length on nurse and patient outcomes remains mixed and context-dependent.

Previous studies have associated long shifts with nurse fatigue, reduced alertness, and increased error rates [1, 2]. At the same time, 12-h shifts have been linked to improved continuity of care and better work-life balance, especially among nurses with family responsibilities [3, 4]. While much of the literature originates from Western or high-income countries, cultural, organizational, and regulatory differences may influence how shift length affects nurses in Oman.

Despite the widespread implementation of extended shifts during the pandemic, no published study has yet examined the impact of 12-h versus 8-h shifts in Oman. While international studies offer mixed findings, the recent research from Oman indicates that approximately 65.6% of nurses experience high levels of burnout, primarily due to factors such as inadequate staffing and limited managerial support. Additionally, night shift duties have been linked to increased work-related stress among Omani nurses. These findings highlight the pressing need to explore the impact of shift lengths on nurses' well-being within the Omani healthcare context [5]. This research seeks to address this gap by comparing nurses' perceptions of fatigue, quality of life (QOL), satisfaction, turnover intention, and patient care outcomes.

## 2. Aim

This study is unique in its context and timing, as it evaluates the shift system during a critical healthcare period in a tertiary Omani hospital. Its findings are intended to inform nurse managers and healthcare policymakers on the implications of shift length within similar healthcare settings.

This study aimed to answer the following research questions:

• Are there significant differences in fatigue, QOL, and job satisfaction between nurses' working 8-h versus 12-h shifts?
• Does shift length influence nurses' intention to leave (ITL) or perceived patient safety incidents?
• Does the type of shift and other variables predict the nurses QOL in Oman?

## 3. Background

The global shortage of nurses, which exceeded 5.9 million even before the COVID-19 pandemic, has pushed healthcare systems worldwide to adopt innovative strategies to maintain the quality and continuity of care. One widely adopted intervention is the restructuring of shift patterns, particularly transitioning from traditional 8-h shifts to 12-h shifts in response to workforce shortages. Numerous studies have investigated the outcomes associated with different shift lengths, especially the 12- and 8-h shifts [1, 6]. While these extended shifts can help reduce handovers and improve continuity of care, they also expose nurses to prolonged physical and psychological stressors such as fatigue, sleep disturbances, and impaired circadian rhythms, which may lead to chronic health conditions [7, 8].

Fatigue has been highlighted as a key concern in the nursing literature due to its adverse effects on job satisfaction, mental well-being, and QOL [9]. The Registered Nurses' Association of Ontario defines fatigue as a subjective state that includes a spectrum from mild tiredness to full exhaustion, compromising both cognitive and physical functioning. However, research findings on the relationship between shift length and fatigue are inconsistent. While some studies demonstrate that 12-h shifts are associated with increased fatigue [10–12], others suggest that such shifts may allow for longer rest periods and better work-life balance. An integrative review by Min et al. [13] further confirms this ambiguity, concluding that fatigue levels among nurses may not be attributed solely to shift length but are also influenced by other scheduling factors such as quick returns and unpredictable shift changes. Beyond fatigue, QOL is another important consideration in evaluating shift systems. Some studies report that 12-h shifts are preferred by nurses due to cost savings, fewer commutes, and increased time for family responsibilities, all of which contribute positively to QOL [1, 14]. However, not all studies agree; for example, Min et al. [13] found no statistically significant differences in QOL, fatigue, or safety incidents between nurses working different shift durations. Despite these mixed results, longer shifts have been associated with improved healthcare outcomes in certain cases by allowing more consistent patient monitoring and reducing missed nursing care [15].

With regard to patient safety and performance, the evidence remains inconclusive. Objective assessments suggest a potential decline in cognitive performance and increase in error rates over successive long shifts [16, 17]. However, a pilot study comparing 8- and 12-h shift nurses found no significant differences in cognitive errors [18]. These findings illustrate the challenge of linking shift length directly to care quality due to the lack of comprehensive and comparative data. While some nurses report improved continuity of care during longer shifts, empirical studies do not consistently confirm a positive impact on patient outcomes [15, 19].

The relationship between shift length and nurses' ITL is similarly complex. Although longer shifts have been promoted as a retention strategy due to perceived job satisfaction and flexibility, several studies have reported that extended working hours are associated with increased job dissatisfaction and higher turnover intention [6, 8, 9, 14, 19]. This reflects the multifactorial nature of turnover, which can be influenced by individual, unit-level, and organizational factors [20].

In summary, the literature reflects considerable variation in how shift length affects nurse and patient outcomes. While some evidence highlights the operational benefits and flexibility associated with 12-h shifts, other studies raise concerns about fatigue, performance, and safety. Notably, few studies have examined these effects in the context of Oman. This study seeks to fill that gap by evaluating the impact of shift length on fatigue, QOL, turnover intention, and perceived care quality among nurses at a tertiary hospital in Oman. A preliminary version of this research was shared as a preprint on Research Square [21] to facilitate early dissemination and feedback. The current version incorporates refinements in the discussion and interpretation of findings, ensuring alignment with the latest evidence and addressing feedback received from initial readers.

## 4. Methods

### 4.1. Design, Setting, and Sample

This study employed a comparative cross-sectional quantitative design and was conducted at SQUH in Oman. A nonprobability convenience sampling approach was used to recruit participants who met the eligibility criteria. While this method allowed efficient recruitment during the COVID-19 pandemic, it may limit the generalizability of findings due to its single-site design.

The required sample size, calculated using G∗Power with a 95% confidence level, α = 0.05, medium effect size (Cohen's *f*^2^ = 0.15), and 80% power, was 120 nurses. Anticipating attrition, the sample size was increased to 132. However, only 100 complete responses were retained for analysis due to survey noncompletion and data quality issues. This reduction slightly lowered the statistical power and represents a limitation. Eligible participants included male and female nurses (Omani and non-Omani) with at least a Diploma in Nursing and a minimum of 1 year of professional experience. Additionally, they were required to have worked in either a 12-h or 8-h shift schedule for at least six months. Nurses who did not meet these criteria—such as those with less than a diploma, less than 1 year of experience, or fewer than 6 months on a set shift schedule—were excluded to maintain data integrity. This sampling strategy ensured the inclusion of participants capable of providing reliability and contextually meaningful responses about the shift system.

### 4.2. Data Collection and Ethical Considerations

The study adhered to ethical standards approved by the Medical Research and Ethics Committee of Sultan Qaboos University (SQU-EC/035/2023, MREC #2965). After securing hospital administrative approval, potential participants were contacted via institutional email. The Participant Information Sheet (PIS) outlined the study's aims, potential risks and benefits, and confidentiality protections. Nurses who consented to participate accessed the survey through a secure online link. While online administration facilitated access, it introduced potential selection bias by favoring those more comfortable with digital tools or more motivated to respond.

The survey took approximately 10–12 min to complete, with reminder emails sent biweekly to encourage participation. Informed consent was obtained electronically, and participation was entirely voluntary. Anonymity and confidentiality were strictly maintained through encrypted data storage and password-protected access limited to the research team.

Informed consent was obtained electronically, and participation was entirely voluntary. Participants were assured of their right to skip any question or withdraw without penalty. To ensure anonymity and confidentiality, no identifiable information was collected, and responses were stored on encrypted, password-protected devices accessible only to the research team. These measures safeguarded participant privacy and upheld the ethical integrity of the research process.

### 4.3. Description of Study Instruments

A total of 93 items were included in the questionnaire. The study used several validated and custom-developed instruments. Demographic characteristics were assessed with seven items. Fatigue was measured using the 20-item Checklist Individual Strength (CIS) scale. QOL was assessed using the 26-item World Health Organizational Quality of Life Brief (WHOQOL-BREF). Turnover intention was measured with the six-item Turnover Intention Scale (TIS-6). Three items captured the frequency of safety incidents.

Custom tools were developed to assess shift justification (five items), willingness to retain shift system (one item), and shift perception (22 items). These were reviewed by an expert panel for face and content validity. Although not subjected to full psychometric testing, they were adapted from prior literature [1] and validated through expert consensus. No linguistic adaptations were necessary as the instruments were administered in English which is the operational language at the study site.

Custom tools, including the shift perception and justification items, were reviewed by a panel of expert nurses to establish face and content validity. These tools were adapted from Hong et al.'s study [1], and expert feedback confirmed cultural appropriateness for the Omani context. Since English is the official working language at SQUH, linguistic translation was not required. Although full psychometric testing was not conducted locally, previous international studies have reported acceptable reliability. Further testing is recommended in future studies.

The first section assessed the demographic information. It included items on participants' age, gender, education level marital status, number of kids, working unit, and total work experience.

Fatigue was assessed using the CIS scale. The CIS is a 20-item survey that asks workers how they have been feeling over the last two weeks in order to quantify many facets of fatigue. Eight questions of CIS assess the subjective feeling of fatigue, five questions measure the concentration, four questions for motivation, and another four to assess degree of physical activity. The whole scale has a 0.93 Cronbach's alpha. Likert scales with seven points were used to score CIS items. Higher ratings signify greater levels of fatigue, more issues with focus, decreased motivation, or less activity. The total score from all subscales range from 20 to 140 [22].

The nurses' QOL was assessed using the WHOQOL-BREF scale. It is a widely used scale which was developed by the World Health Organization (WHO) [23] and had been translated to more than 26 languages. It consists of a total of 26 items where the answer options for each item range from 1 which represents *very dissatisfied/poor* to 5 which represents *extremely satisfied/excellent*. The scale assessed the quality of four aspects of nurse life: physical wellbeing, mental wellbeing, social interactions, and the environment. The scores are transformed into a linear scale with 0 being the most unfavorable and 100 being the most favorable, ranging from 0 to 100. The scale has been widely used and its psychometric properties established to show satisfactory validity and reliability with Cronbach's alpha range between 0.60 and 0.90 [23].

The turnover intention was assessed by TIS-6 which was developed by Bothma and Roodt [24]. It comprises six item, and subjects were responded to each item by selecting one option between 1 and 5, where 1 (*not at all*) and 5 (*strongly agree*), with summation of scores greater than 18 indicate an overall ITL, whereas scores less than 18 indicate intention to stay. The scale has been widely used and its psychometric properties established to show satisfactory validity and reliability with Cronbach's alpha for it is 0.80 [24].

To determine the frequency of safety incidents, three items were used. These inquired about the occurrence and frequency of medication errors that resulted in both dangerous and benign results, as well as the occurrence and rate of medication errors that almost resulted in a needlestick injury in the preceding 2 months. The frequency of needlestick accidents, patient fall incident, medication mistakes, and near-miss medication errors were reported by the respondents. This item was developed by Hong et al. [1] and has been utilized previously.

A five-item questionnaire was used to examine the reasons for choosing a shift system. Based on their clinical expertise and prior use, expert nurses created the assessment tool. Another expert committee analyzed the major components to confirm their accuracy. They marked the reason(s) to select their current shift length. The given reasons include adequate rest, increased time for self-development and hobbies, child rearing, diminished burden of commuting, and others. Participants were allowed to choose more than one reasons [1]. The willingness to maintain the current shift length was measured using one dichotomous item where subjects choose yes or no as answer. The nurses' perception of the shift system was evaluated using participant responses to a 22-item questionnaire created by Hong et al. [1]. The validity of the tool was established by expert review. The perception was assessed by 22 items which examined the impact of shifts on their personal life (12 items) and nursing practice (10 items).

### 4.4. Data Analysis

Prior to analysis, data were screened for completeness and normality using histograms and the Shapiro–Wilk test. No missing data were found. Assumptions for regression (normality, linearity, and homoscedasticity) were met. Multicollinearity diagnostics showed acceptable VIFs (< 2), indicating no collinearity issues.

Descriptive statistics summarized participant characteristics. Independent *t*-tests and chi-square tests assessed differences across shift types. Effect sizes (Cohen's d and Cramer's V) and 95% confidence intervals were reported. Multiple linear regression was conducted to identify predictors of QOL, adjusting for shift type, age, gender, marital status, fatigue, satisfaction, and unit assignment. The data were analyzed using the Statistical Packages for Social Sciences (SPSS, Version 25). Descriptive statistics were computed to summarize the demographic and professional characteristics of the sample, including frequencies, percentages, means, and standard deviations. To assess differences between nurses working 8- and 12-h shifts, independent *t*-tests were conducted on fatigue, QOL, satisfaction with shift, and ITL, while chi-square tests were used to evaluate associations between shift type and reported safety incidents. To control for potential confounders and enhance the robustness of the findings, a multiple linear regression analysis was also conducted to examine predictors of QOL, adjusting for variables such as shift type, age, gender, marital status, satisfaction, fatigue, and unit assignment.

## 5. Result

### 5.1. Sample Demographics

Based on the data provided in Table 1, the current working shift for the sample of 100 participants consisted of 70 individuals (70%) working 12-h shifts and 30 individuals (30%) working 8-h shifts. Based on the descriptive statistics provided, the sample of 100 participants had an average age of 31.58 years (SD = 5.824). Based on Table 2, the participants' age ranged from 21 years to 53 years. In terms of total working experience in years, the participants had an average of 7.47 years (SD = 5.638), ranging from 1 to 24 years of experience. Furthermore, the majority of participants were female (74%), with 26% male. In terms of education level, 88% of participants held a Bachelor's degree in nursing, while 8% held a Diploma in Nursing and 4% held a Master's degree. The majority of participants were married (65%), with 31% single and 4% divorced. The working units of the participants were also varied, with the largest group working in the medical unit (28%), followed by surgical (25%), emergency department (15%), pediatric (15%), ICY (9%), and OPD (8%).

### 5.2. Bivariate Comparisons by Shift Type

Significant differences were found between shift groups across several variables: nurses on 12-h shifts reported higher satisfaction (*M* = 3.79, SD = 0.93) than those on 8-h shifts (*M* = 3.03, SD = 1.30), *t*(98) = 3.27, *p*=0.001. They also had higher fatigue levels (*M* = 3.72 vs. 2.85), *t*(98) = 3.82, *p* < 0.001, and higher QOL scores (*M* = 3.20 vs. 2.58), *t*(98) = 4.20, *p* < 0.001. No significant difference was observed in ITL, *t*(98) = 0.17, *p*=0.864.

### 5.3. Safety Incident Associations

In Table 3, chi-square analysis showed no association between shift type and needlestick injuries (*χ*^2^(1) = 0.38, *p*=0.540), medication errors (*χ*^2^(1) = 0.53, *p*=0.467), or near-miss medication errors (*χ*^2^(1) = 0.67, *p*=0.413). However, patient falls were significantly more frequent among nurses working 12-h shifts, *χ*^2^(1) = 8.52, *p*=0.004. There was also a significant difference in nurses' willingness to continue their current shift systems, *χ*^2^(2) = 9.66, *p*=0.008.

### 5.4. Regression Analysis for Predictors of QOL

To explore predictors of QOL while accounting for confounders, a multiple linear regression was conducted, and the result is illustrated in Table 4. A multiple regression model predicting QOL was significant (*F*(16, 83) = 9.64, *p* < 0.001, adjusted *R*^2^= 0.583). Satisfaction with shift was the strongest positive predictor (*β* = 0.432, *p* < 0.001, 95% CI [0.117, 0.287]), while working a 12-h shift had a significant negative association with QOL (*β* = −0.232, *p*=0.004, 95% CI [−0.603, −0.117]). Marital status showed a marginally significant positive effect (*β* = 0.180, *p*=0.051, 95% CI [−0.001, 0.555]). while other variables—including fatigue, age, gender, education level, and specific work units—did not significantly predict QOL.

### 5.5. Nurses' Perceptions of Shift Systems

Participants also shared their perceptions of the advantages and disadvantages of their current shift systems. Nurses working 12-h shifts identified benefits such as increased rest time, continuity of care, and more opportunities for personal development and childcare, with 86% citing rest as the primary benefit. However, they also reported disadvantages including greater physical and emotional exhaustion, difficulty adapting to policy changes, decreased concentration, and increased risks to patient safety. On the other hand, nurses working 8-h shifts noted reduced physical fatigue, improved communication with patients and staff, and better patient relationships. However, they also faced challenges in managing time for care planning and participating in departmental meetings and educational activities.

## 6. Discussion

This study explored the comparative effects of 8-h versus 12-h shifts on nurse fatigue, QOL, perceived patient safety, and turnover intention within a tertiary hospital in Oman. As the global nursing workforce grapples with evolving work-hour models, these findings offer critical context-specific insights into a longstanding and complex debate. While several key themes align with the international literature, this study contributes uniquely to the regional discourse on nursing shift patterns, especially in Middle Eastern healthcare systems where such data are limited [19].

One of the most consistent and significant findings was the elevated fatigue reported by nurses working 12-h shifts. This result is congruent with prior studies that link longer working hours to cumulative mental and physical exhaustion. Nurses on extended shifts often experience circadian disruption, insufficient recovery time between shifts, and limited opportunities for rest breaks which are factors known to heighten fatigue levels [1, 7, 11]. However, the broader literature remains inconclusive; Bae and Fabry [25] found no significant differences, suggesting that fatigue may be moderated by contextual variables such as workload intensity, availability of breaks, or shift scheduling consistency. Notably, this study did not control for patient acuity or unit workload, which may influence fatigue outcomes and should be examined in future research. Furthermore, Jarrar et al. [7] emphasized that organizational culture and leadership styles also influence nurse well-being, which may explain some of the variation across settings. Notably, this study did not control for patient acuity or unit workload, which may influence fatigue outcomes and should be examined in future research.

Interestingly, despite higher fatigue, nurses working 12-h shifts reported better QOL, potentially due to increased rest days and improved work-life integration. Nonetheless, the QOL improvement should be interpreted cautiously given concurrent reports of increased patient falls and fatigue. It is possible that nurses value autonomy and consecutive days off, which may offset perceived physical strain. Cultural factors such as familial support structures and public sector job stability in Oman may also shape these perceptions, reinforcing the need for qualitative inquiry[3, 4]. These findings are in line with Jarrar et al. [7], who reported that while longer shifts increased fatigue, they also improved perceived autonomy and continuity of care, indirectly supporting work-life balance. This reinforces the need for holistic assessment tools that capture the multifaceted experiences of nurses rather than solely clinical indicators. These insights were further substantiated through regression analysis, where overall satisfaction with the current shift was the strongest predictor of QOL. In contrast, fatigue, while negatively associated, was not a statistically significant predictor, suggesting that nurses may perceive their overall well-being through a broader lens that extends beyond momentary physical tiredness. Furthermore, the model explained a substantial portion of the variance in QOL, strengthening the credibility of these associations.

Perceptions of safety were mostly comparable between groups, except for patient falls, which were more common among those on longer shifts. While continuity of care may be improved with fewer handovers, vigilance toward the end of long shifts may decline, increasing safety risks. This aligns with the dual perspective in the literature: while longer shifts reduce handovers and may enhance continuity of care [1], they may also impair alertness and cognitive performance toward the end of shifts [4]. Falls are often linked to delayed response times and inattentiveness, which could explain this finding. Jarrar et al. [19] similarly reported that prolonged duty hours were associated with a higher risk of adverse events, reinforcing the need for better fatigue management strategies. Therefore, nursing managers should consider targeted strategies, such as increased rounding or scheduled microbreaks during longer shifts, to mitigate risks.

No significant difference in turnover intention was observed, but this could reflect cultural and organizational influences unique to the Omani context, such as limited mobility or job security in government roles. Nurses' free-text responses further illuminated the nuanced trade-offs between shift durations, highlighting the value of rest and autonomy on one hand and concerns over fatigue and safety on the other. Previous studies [9, 14, 26] suggest that prolonged exposure to 12-h shifts can accumulate dissatisfaction and lead to attrition over time. Jarrar et al. [14] particularly highlighted that ill-being and perceived organizational support played a critical role in nurses' ITL, indicating that factors beyond shift length alone may influence retention. The cross-sectional nature and relatively short time since the shift system's implementation may have limited this study's ability to detect longer-term patterns. Moreover, cultural factors in Oman, such as job stability in the public sector or familial support structures, may moderate the effects of shift length on ITL, warranting further qualitative investigation. Nurses' qualitative responses illuminated the broader implications of shift work on personal life and professional responsibilities. Those working 12-h shifts emphasized benefits such as increased rest time and greater flexibility for personal pursuits, especially for those with caregiving duties. However, they also expressed concerns about heightened physical and emotional strain, difficulty maintaining concentration, and increased safety risks. Conversely, nurses on 8-h shifts reported stronger interpersonal relationships with patients and coworkers but struggled with fragmented rest and insufficient time for nursing care planning. These findings underscore the nuanced trade-offs between shift lengths and support a personalized approach to scheduling. These findings highlight the complex relationship between shift length and nursing outcomes. The high prevalence of burnout among Omani nurses, as evidenced by recent studies, suggests that extended shift lengths may exacerbate existing stressors. Furthermore, regional data from gulf countries, such as the 65.4% burnout rate among emergency department nurses in Saudi Arabia, reinforce the need for context-specific strategies to mitigate burnout and improve nurse retention [27].

From a practice standpoint, 12-h shifts were linked to perceived improvements in care continuity, while 8-h shifts were associated with better communication and fewer safety concerns. Both perceptions have empirical support [14, 28], yet the diversity in responses indicates that unit-specific demands, staff characteristics, and institutional culture heavily shape these outcomes. The evidence suggests that one-size-fits-all scheduling policies may be ineffective; rather, a flexible, individualized approach to shift design aligned with local workforce dynamics and cultural preferences.

## 7. Limitations

This study has several limitations. First, the convenience sample from a single tertiary hospital in Oman limits generalizability. Although a sample size of 132 was targeted, only 100 complete responses were analyzed due to survey incompletion and data quality checks. The cross-sectional design precludes causal interpretations.

Importantly, the study did not control for critical confounders such as workload intensity, nurse-to-patient ratios, or patient acuity, which could have influenced both fatigue and safety incident outcomes. Additionally, reliance on self-reported data introduces the potential for recall and social desirability bias. Despite these limitations, the study provides timely, context-specific insights and lays a foundation for future longitudinal research.

## 8. Implications for Research and Practice

This study provides actionable insights for both nursing management and future research. From a practice perspective, hospital administrators should consider adopting flexible shift policies that account for the diverse needs and preferences of nursing staff. Given that shift satisfaction emerged as a critical predictor of QOL, enabling nurses to have some autonomy in scheduling may improve overall well-being and work engagement. Strategies such as scheduled rest breaks, optimized handover protocols, and regular wellness check-ins may mitigate the negative effects of fatigue, especially for those on longer shifts.

In terms of policy, healthcare institutions in Oman and similar contexts may benefit from data-driven staffing policies that integrate shift satisfaction and performance metrics. Incorporating nurse feedback into shift design can foster greater morale and retention, which are essential amid ongoing workforce shortages.

For future research, longitudinal studies are needed to examine how prolonged exposure to different shift models impacts burnout, job satisfaction, and clinical performance over time. Mixed-methods research that includes qualitative interviews may offer deeper insight into contextual or cultural moderators that influence the perceived benefits and drawbacks of shift length. Additionally, studies comparing different specialties or levels of nurse experience could help refine shift policies that align more closely with specific clinical roles.

## 9. Conclusion

This study contributes valuable evidence on the impact of 8-h and 12-h shifts in the Omani healthcare context. While 12-h shifts were linked to higher fatigue and patient fall rates, they were also associated with better perceived QOL and shift satisfaction. These outcomes likely reflect trade-offs between rest, autonomy, and work-related strain.

Importantly, shift satisfaction emerged as the strongest predictor of QOL, highlighting the need to align scheduling with nurses' preferences and needs. However, the increased fatigue and fall rates suggest caution in generalizing support for extended shifts.

Healthcare managers and policymakers are encouraged to implement flexible and individualized shift policies rather than adopting one-size-fits-all models. Longitudinal and multisite studies are needed to assess the long-term consequences of shift configurations on nurse retention, safety, and care quality. These findings affirm the need for healthcare institutions to avoid binary decisions about shift length. Instead, they should strive to create adaptable shift systems that promote both organizational efficiency and nurse well-being. Administrators should consider policies that allow shift flexibility, ensure adequate recovery time, and incorporate nurses' preferences into scheduling decisions. Further longitudinal and multisite studies are essential to explore the long-term consequences of shift models on retention, safety, and overall care quality.

## Figures and Tables

**Table 1:** Demographic characteristics (*N* = 100).

		Frequency	Percent	Mean	SD
Gender	Female	74	74.0		
	Male	26	26.0		

Education level	Bachelor's degree in nursing	88	88.0		
	Diploma in nursing	8	8.0		
	Master's degree and above	4	4.0		

Marital status	Divorced/widowed	4	4.0		
	Married	65	65.0		
	Single	31	31.0		

Having children	No children	35	35.0		
	Less than 3	44	44.0		
	3 and more	21	21.0		

Working unit	Emergency department	15	15.0		
	ICU	9	9.0		
	Medical	28	28.0		
	OPD	8	8.0		
	Pediatric	15	15.0		
	Surgical	25	25.0		

Age				31.58	5.82

Working experience				7.47	5.63

Abbreviations: ICU = Intensive Care Unit and OPD = outpatient department.

**Table 2:** Comparison of key variables by shift length (*N* = 100).

	*t*	df	Sig. (2-tailed)	Mean difference	Std. error difference	95% confidence interval of the difference	
						Lower	Upper
Satisfaction with the current shift	3.273	98	0.001	0.752	0.230	0.296	1.209
Fatigue level	3.821	98	0.000	0.873	0.228	0.419	1.326
QOL	4.201	98	0.000	0.625	0.149	0.330	0.921
ITL	0.171	98	0.864	0.024	0.143	−0.259	0.307

Abbreviations: ITL = intention to leave and QOL = quality of life.

**Table 3:** Safety incidents and shift preferences (*N* = 100).

		12-h shift	8-h shift	*χ* ^2^	df	*p*
Needlestick accident	Yes	18	6	0.376	1	0.540
	No	52	24			

Medication errors	Yes	8	2	0.529	1	0.467
	No	62	28			

Near-miss medication errors	Yes	27	9	0.670	1	0.413
	No	43	21			

Patient fall	Yes	36	6	8.515	1	**0.004**
	No	34	24			

Are you willing to continue the current shifting system?	Yes	54	14	9.656	2	**0.008**
	No	6	4			
	Maybe	10	12			

*Note:* Bold values are significant at 0.05.

**Table 4:** Regression coefficients predicting QOL.

	Unstandardized coefficients		Standardized coefficients	*t*	Sig.	95.0% confidence interval	
	*B*	Std. error	Beta			Lower bound	Upper bound
(Constant)	3.649	0.549		6.650	**0.000**	2.557	4.740
Fatigue level	−0.055	0.061	−0.065	−0.890	0.376	−0.177	0.067
Satisfaction with the current shift	0.202	0.043	0.432	4.707	**0.000**	0.117	0.287
Age	−0.024	0.013	−0.187	−1.882	0.063	−0.049	0.001
Shift type (reference: 8-h shift)							
12-h shift	−0.360	0.122	−0.232	−2.942	**0.004**	−0.603	−0.117
Gender (reference: female)							
Male	0.095	0.118	0.057	0.807	0.422	−0.139	0.329
Educational level (reference: Master's degree and above)							
Diploma in nursing	0.074	0.340	0.027	0.217	0.829	−0.603	0.751
Bachelor's degree in nursing	−0.168	0.270	−0.074	−0.623	0.535	−0.705	0.369
Marital status (reference: single)							
Married	0.277	0.140	0.180	1.984	**0.051**	−0.001	0.555
Divorced/widowed	0.494	0.293	0.132	1.683	0.096	−0.090	1.077
Working unit (reference: medical)							
Surgical	0.253	0.139	0.149	1.821	0.072	−0.023	0.529
OPD	−0.205	0.120	−0.135	−1.707	0.092	−0.443	0.034
Emergency department	−0.280	0.151	−0.190	−1.857	0.067	−0.579	0.020
ICU	−0.034	0.188	−0.013	−0.181	0.857	−0.409	0.340
Pediatric	0.153	0.158	0.074	0.969	0.335	−0.161	0.467
Willingness to continue working the same shift (reference: willing to continue)							
Not willing to continue	−0.034	0.180	−0.015	−0.191	0.849	−0.392	0.323
Not sure	0.120	0.119	0.074	1.005	0.318	−0.118	0.358

*Note:* Bold values are significant at 0.05.

Abbreviations: ICU = Intensive Care Unit and OPD = outpatient department.

## Data Availability

The data that support the findings of this study are available on request from the corresponding author. The data are not publicly available due to privacy or ethical restrictions.
